# Transcriptome analysis of the venom gland of the Mexican scorpion *Hadrurus gertschi *(Arachnida: Scorpiones)

**DOI:** 10.1186/1471-2164-8-119

**Published:** 2007-05-16

**Authors:** Elisabeth F Schwartz, Elia Diego-Garcia, Ricardo C Rodríguez de la Vega, Lourival D Possani

**Affiliations:** 1 Departamento de Medicina Molecular y Bioprocesos, Instituto de Biotecnología, Universidad Nacional Autónoma de México, Avenida Universidad, 2001 Cuernavaca 62210, Mexico; 2 Departamento de Ciências Fisiológicas, Instituto de Ciências Biológicas, Universidade de Brasília, Brasília, DF, 70910-900, Brasil

## Abstract

**Background:**

Scorpions like other venomous animals posses a highly specialized organ that produces, secretes and disposes the venom components. In these animals, the last postabdominal segment, named telson, contains a pair of venomous glands connected to the stinger. The isolation of numerous scorpion toxins, along with cDNA-based gene cloning and, more recently, proteomic analyses have provided us with a large collection of venom components sequences. However, all of them are secreted, or at least are predicted to be secretable gene products. Therefore very little is known about the cellular processes that normally take place inside the glands for production of the venom mixture. To gain insights into the scorpion venom gland biology, we have decided to perform a transcriptomic analysis by constructing a cDNA library and conducting a random sequencing screening of the transcripts.

**Results:**

From the cDNA library prepared from a single venom gland of the scorpion *Hadrurus gertschi*, 160 expressed sequence tags (ESTs) were analyzed. These transcripts were further clustered into 68 unique sequences (20 contigs and 48 singlets), with an average length of 919 bp. Half of the ESTs can be confidentially assigned as homologues of annotated gene products. Annotation of these ESTs, with the aid of Gene Ontology terms and homology to eukaryotic orthologous groups, reveals some cellular processes important for venom gland function; including high protein synthesis, tuned posttranslational processing and trafficking. Nonetheless, the main group of the identified gene products includes ESTs similar to known scorpion toxins or other previously characterized scorpion venom components, which account for nearly 60% of the identified proteins.

**Conclusion:**

To the best of our knowledge this report contains the first transcriptome analysis of genes transcribed by the venomous gland of a scorpion. The data were obtained for the species *Hadrurus gertschi*, belonging to the family Caraboctonidae. One hundred and sixty ESTs were analyzed, showing enrichment in genes that encode for products similar to known venom components, but also provides the first sketch of cellular components, molecular functions, biological processes and some unique sequences of the scorpion venom gland.

## Background

Scorpion venoms are very complex mixtures with hundreds of different components produced by the highly specialized venom glands. The most prominent components of scorpion venoms are the peptides responsible for the neurotoxic effects associated with their sting, for which more than 350 different have been described (extensive databases can be found in Tox-Prot [1] and SCORPION [2]). Most of these toxins are structurally related disulphide-rich short proteins (23–75 amino acid residues long), which affect cellular communication by modulating Na^+ ^or K^+ ^ion-channels permeability [3]. Due to their importance in scorpion envenomation and their usefulness as molecular and pharmacological probes for studying ion-channels, most of the work performed to date are focused at these neurotoxins, with relative few other components ever described; among which are heterodimeric phospholipases A2 (*v.gr. *[4-6]), non-disulphide short peptides with cytolytic activity and a few other functions [7,8]. Recent proteomic analyses [9-16] have documented the overall composition for nine scorpion species, all of them from the family Buthidae and most of them belonging to the *Tityus *genus. These analyses confirmed the gross estimation of an average of one hundred different proteins in each one of the venoms [17]. Approximately half of them comprehend components with molecular masses in the range of commonly found scorpion toxins (2,000–8,000 Da). These numbers contrast heavily with the known universe of protein components (near four hundreds) described to exist in scorpion venoms, from which only about 12% are not classified within the known scorpion toxin families.

Further insights into scorpion venom compositions have been achieved by gene cloning by PCR-based methods conducted with cDNA libraries. For example, almost one hundred toxin precursors have been sequenced from venom gland libraries of the buthid scorpion *Mesobuthus martensii *(*v.gr. *[18-20]). Unfortunately the spectrum of sequences obtained through PCR-based approach is limited by the specificity of the PCR primers used. It is worth noticing that although PCR-based methods along with the abundant isolation and characterization of scorpion toxins and, more recently, proteomic profiling of whole venoms, have provided us with a large number of sequences, all these components are secreted from the venom glands. Little is known about the biological processes that are taking place inside the venom gland cells. Therefore, we elected to use a transcriptome approach to improve the understanding of the composition of *Hadrurus gertschi *venom gland.

The scorpion *H. gertschi *Soleglad (1976) belongs to the family Caraboctonidae [21] and is considered no dangerous to humans. *H. gertschi *is endemic to Mexico, occurring exclusively in the State of Guerrero, and lives underground in tunnels excavated in the soil. From the venom of this scorpion few components have been isolated and studied: hadrurin, an antimicrobial and cytolytic peptide [22]; HgeTx1, a K^+ ^channel blocker [23]; hadrucalcine, a peptide capable of activating skeletal Ryanodine receptors [Schwartz et al., in preparation], and; the precursors HgeScplp and HgeβKTx, which encode for long-chain peptides similar to Scorpine and βKTx's, respectively [24]. Although hadrurin was reported as component of *H. aztecus *venom [22], the specimens used in that work were not taxonomically identified and latter it was realized that scorpions from that geographical region should be named *H. gertschi*; this species assignment was confirmed by identification of relevant taxonomic keys in the specimens.

In the present work we randomly generated and analyzed 160 expressed sequence tags (ESTs) from a cDNA library of the venom gland of *H. gertschi*. These 160 ESTs corresponded to 0.15% of the whole non-amplified cDNA library and were generated from a non-normalized cDNA library. After clustering the resulting dataset, we identified transcripts possibly associated with different cellular functions. The possible roles of some of the transcripts are discussed, although many have unknown functions. Furthermore, we present 8 full length sequences of new toxins.

Single-pass gene sequencing from cDNA libraries is an affordable strategy to mine the transcript profile of a given tissue [25]. This strategy has been used to analyze the transcripts profiles for few other venomous organisms, such as cnidarians [26,27], cone snails [28], fishes [29], snakes (*v.gr. *[30-32]) and spiders [33]. To the best of our knowledge, this is the first report of an ESTs strategy conducted with any scorpion venom gland. Moreover, this is the first comprehensive molecular study of a non-buthid scorpion, which could serve for comparative purposes when studying the details of the process by which buthid scorpions have been assembling their neurotoxic arsenal.

## Results

### cDNA library and EST analysis

**Figure 1:**
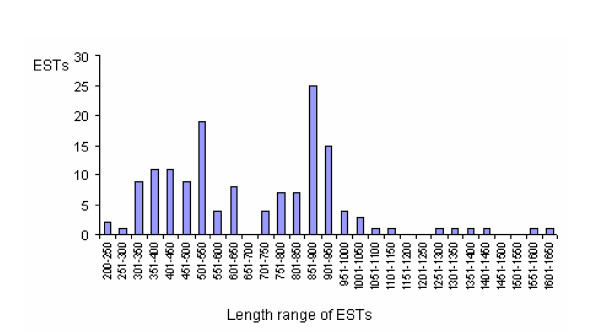
**Reads length distribution of *H. gertschi *venom gland ESTs**. A total of 147 ESTs were analyzed in the current study. Abscissa is the length of sequences in 50 bp intervals, whereas the total number of ESTs for each cluster is shown in the Y-coordinate.

The *H. gertschi *venom gland library constructed was not amplified (2.8 × 10^5 ^cfu/mL with 99% recombinant clones); therefore the cluster size might reflect the relative abundance of the corresponding mRNA population (see [34,35], but also [36,37]). After sequencing, 160 electropherograms were submitted to bioinformatics analysis to remove vector and poor quality sequences, resulting in 147 high-quality ESTs which were used to analyze gene expression profile in the *H. gertschi *venom glands. The mean read length of ESTs was 919 nucleotides (ranging from 225 to 1613 nucleotides, Figure 1). After clusterization 20 clusters showing more than one EST and 48 singlets were grouped (Table 1). Among the 147 ESTs, in 29% (4 contigs and 19 singlets) we were unable to identify any open reading frame (ORF). The remaining sequences encode for protein precursors with an average of 131 residues (from 66 to 285 amino acid residues long). The complete dbEST submission with 68 nucleotide sequences and annotations is included in Additional file 2 as raw text.

**Table 1:** Identification of the transcripts predicted to be involved in common cellular processes and those similar to known venom components. The putative identity corresponds to the eukaryotic orthologous group (KOG), as detailed in Methods.

**Sequence Id**	**GenBank**	**Descriptor**
Gene products predicted to be involved in common cellular processes		
HGE001|Contig13	EL698878	KOG1376 Alpha tubulin
HGE003|Contig21	EL698880	KOG3412 60S ribosomal protein L28
HGE004|Contig12	EL698881	KOG0279 G protein beta subunit-like protein
HGE005|Contig19	EL698882	KOG1954 Endocytosis/signaling protein EHD1
HGE006|2203	EL698883	KOG3418 60S ribosomal protein L27
HGE007|2225	EL698884	KOG3449 60S acidic ribosomal protein P2
HGE008|2233	EL698944	KOG0714 Molecular chaperone (DnaJ superfamily)
HGE009|2404	EL698885	KOG3458 NADH:ubiquinone oxidoreductase, NDUFA8/PGIV/19 kDa subunit
HGE010|2258	EL698886	KOG0863 20S proteasome, regulatory subunit alpha type PSMA1/PRE5
HGE011|2268	EL698887	KOG3311 Ribosomal protein S18 (40S)
HGE012|2330	EL698888	KOG1629 Bax-mediated apoptosis inhibitor TEGT/BI-1
HGE013|2397	EL698889	KOG0898 40S ribosomal protein S15
HGE014|2453	EL698890	KOG2597 Predicted aminopeptidase of the M17 family
HGE015|2217	EL698891	KOG3752 Ribonuclease H
HGE017|2209	EL698945	KOG0876 Manganese superoxide dismutase
HGE018|2232	EL698892	KOG2941 Beta-1,4-mannosyltransferase
HGE020|2328	EL698894	KOG4075 Cytochrome c oxidase, subunit IV/COX5b
HGE021|2448	EL698895	KOG2667 COPII vesicle protein
HGE022|contig17	EL698896	KOG2403 Succinate dehydrogenase, flavoprotein subunit
HGE023|2323	EL698897	KOG2486 Predicted GTPase
HGE033|2208	EL698907	KOG4604 Uncharacterized conserved protein
Gene products similar to known venom components		
HGE024|Contig2	EL698898	α-KTx 6 subfamily
HGE025|Contig5	EL698899	Novel α-KTx
HGE034|Hgscplike1	EL698908	Scorpine-like group
HGE026|Hgscplike2	EL698900	Scorpine-like group
HGE035|HgbetaKTx1	EL698909	Novel β-KTx
HGE027|NDPB_5.5	EL698901	Novel NDBP group 5
HGE028|NDPB_5.6	EL698902	Novel NDBP group 5
HGE029|NDPB_3.7	EL698903	Novel NDBP group 3
HGE031|PLA2	EL698905	Novel group III heterodimeric phospholipase
HGE030|Hg1	EL698904	KOG4295 Serine proteinase inhibitor (KU family)

### Similarity searches and sequence annotation

All sequences were submitted for blastn and blastx searches against nr database; an e-value < 10^-5 ^was used as cut-off for confidential homologue detection. In addition to the ESTs for which no clear ORF have been identified, nearly 19% of the new sequences (in 3 contigs and 10 singlets) do not match with any entry in the database. Altogether, unassigned ESTs account for close to 48% of the total dataset, a value similar to other transcriptome studies which show values varying from 13% to 56% of non matched sequences [26-33,35]. Noteworthy, these putative gene products signify a source of new information about scorpion venom gland specific genes. In addition to ESTs with no database match, 2 reads presented identity with sequences that have been already described but with no functional assessment, hereby named unknown proteins. One of these, HGE032|2273, is the clone which codes for a protein similar to the CG9896-like proteins identified in the scorpions *Mesobuthus gibbosus *and *M. cyprius*. The other one, HGE033|2208, encodes for a scorpion homologue of short proteins conserved in eukaryotic organisms (KOG4604, pfam04418.6), but whose function is not known yet.

**Figure 2:**
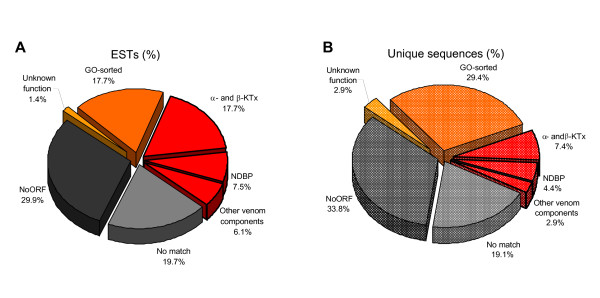
**Relative proportion of each category of the transcripts from *H. gertschi *venom gland library**. A) Relative proportion of each category of the 147 total transcripts from *H. gertschi *venom gland. B) Relative proportion of the unique sequences (20 contigs and 48 singlets). "Unknown function" includes ESTs that presented identity with already described sequences with no functional assessment. "NoORF" includes sequences with non identified open reading frame. "No match" includes ESTs that did not match with currently known sequences. "GO-sorted" includes transcripts coding for proteins involved in cellular processes. "α and β-KTx" transcripts encode for putative K^+ ^toxins from α and β-families, respectively. "NDBP" comprises non-disulfide-bridged peptides. "Other venom components" includes both H. gertschi PLA2 and the Kunitz-type serine proteinase inhibitor.

The identified putative proteins (50% of the total) were assorted into two main groups (Table 1 and Figure 2): 1) precursors similar to gene products implicated in common cellular processes account for 18% of transcripts (in 5 contigs and 15 singlets) and; 2) putative toxins or other venom components, representing 31% of total ESTs (in 8 contigs and 2 singlets). For most of these sequences, the putative identity deposited into the dbEST correspond to the eukaryotic orthologous group (KOG [38]) each would belong (see Materials and Methods), with relevant Gene Ontology (GO [39]) terms assigned with the aid of AmiGO and GOblet [40] web servers.

It is worth noticing that in the group of identified proteins, toxins account for 59.7% of the transcripts (30% of the unique sequences). The distribution of all ESTs is depicted in Figure 2, it can be observed that ESTs coding for toxins are well represented in the *H. gertschi *venom gland transcriptome. Further, considering the non-matched ESTs as a possible source for new toxins, it can be assumed that these molecules are preferentially expressed over proteins related with the other cellular functions.

### GO-sorted annotated sequences

**Figure 3:**
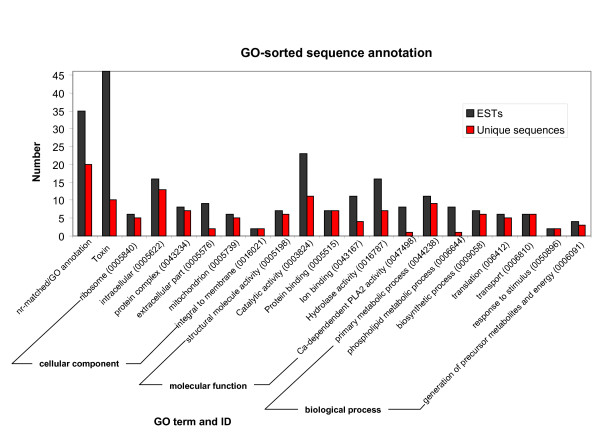
**Gene Ontology-sorted sequence annotation**. Functional classification of all nr-matched transcripts from the *H. gertschi *venom gland. The vertical axis shows the relative proportion of ESTs. The abscissa shows the categories within each of three ontologies: cellular component, molecular function and biological processes. For comparison, the relative proportion of toxin-like ESTs is also shown. All toxin-like sequences were assigned to the special set of the "biological process" ontology called "multi-organism process" (GO:0051704).

All non-toxin nr-matched gene products were annotated in each of the three ontologies of GO: cellular component (CC), molecular function (MF) and biological processes (BP). Within each of these ontologies the categories with highest prevalence are: "intracellular" (11% of total ESTs and 19% of unique sequences), "ribosome" (4.1% and 7.4%), "mitochondrion" (4.1% and 7.4%) and "extracellular part" (6.1% and 2.9%) within CC; "catalytic activity" (16% of total ESTs and unique sequences), "hydrolase activity" (10.9% and 10.3%), "protein binding" (4.8% and 10.3%) and "ion binding" (7.5% and 5.9%) within MF, and; "primary metabolic process" (7.5% of total ESTs and 13.2% of unique sequences), "biosynthetic process" (4.8% and 8.8%), "transport" (4.1% and 8.8%) and "translation" (4.1% and 7.4%) within BP (Figure 3).

## Discussion

### Transcriptome analysis suggest cellular processes relevant for scorpion venom glands function

Although our sampling of the venom glands library is still incomplete, the diversity and nature of the annotated transcripts provide the first glimpse about molecular processes taking part in the scorpion venom gland cells. Since we constructed a non-amplified library, it could be expected that clone number reflects the actual prevalence of a given transcript. Moreover, by extension, different transcripts belonging to the same – confidentially assigned – GO category might suggest this category as important within the biological processes of scorpion venom glands.

For example, intuitively, the venom glands should support high protein synthesis and secretion in order to produce the large amounts of, secreted and renewable, venom proteins. In concordance, 8.2% of the total transcripts and 16.2% of the unique sequences match with either ribosomal components (1 contig and 4 singlets) or proteins involved in cellular trafficking (2 contigs and 1 singlet). Both processes are energetically costly and, consistently, 4.1% of whole ESTs and 7.4% of identified protein precursors match with components of the energy-producer organelle mitochondrion, whereas 2.7% and 4.4%, respectively, are putative homologues of proteins directly involved in the energy-producer oxidative phosphorilation or tricarboxilyc acid cycle. Indeed, scorpions whose venoms were artificially depleted shows increased oxygen consumption [41].

The importance of correct protein processing in the context of scorpion venom gland is emphasized by the presence of transcripts encoding for proteins involved in correct folding (HGE008|2233), posttranslational processing (HGE014|2453 and HGE018|2232) or proteasome-dependent degradation of proteins (HGE010|2258). One of these (HGE014|2453), match with aminopeptidases of the M17 family (KOG2597), which are exopeptidases involved in the processing and regular turnover of intracellular proteins, although their precise role in cellular metabolism is unclear. In particular aminopeptidases of the M17 family cleave leucine residues from the N-terminal of polypeptide chains, but substantial rates are evident for all amino acids [42]. We predicted, by SignalP 3.0, its signal peptide-processing sequence (VAS-LK) suggesting that this transcript is secreted as a venom component, and indeed it could be important for posttranslational modifications of venomous gland components. Moreover, HGE018|2232 match with the glycosylating enzymes β-1,4-mannosyltransferases (KOG2941); which is consistent with the presence of glycosylated proteins in scorpion venoms (*v.gr. *[6]).

An interesting finding in our database was a transcript encoding the ribonuclease H domain of a non-LTR retrotransposon (nLTRrt) of clade R1 (*sensu *[43]). This nLTRrt clade is usually found inserted into telomeres and has been identified in several arthropods (including one arachnid) and some fungi [44]. It is worth noticing that mobile elements and their remnants account for large proportions of most eukaryotic genomes, in which they have had central roles in genome evolution and hypervariation. The expression of transposases indicates that mobile elements might contribute to the diversification of venom toxins. Recently, Glushkov *et al.*, [45] reported the search for nLTRrt in 22 scorpion species, in which degenerate oligonucleotides based on consensus sequences of seven clades of nLTRrt were used to a PCR-based fishing approach. Unfortunately, even though these authors reported that PCR products of the expected size where obtained with R1-based degenerate primers, they only presented data for CR1, I and Jockey nLTRrt clades.

### Toxins and other venom components

Our *H. gertschi *scorpion venom gland library is clearly enriched on toxin-like sequences, with more than 17% (in 4 contigs and 1 singlet) of the sequenced ESTs being similar to known families of scorpion toxins. Another 14% of the total ESTs (in 4 contigs) encodes for precursors which are homologues of previously characterized non-toxin scorpion venom components (see below). Considering that all these sequences contain putative signal peptides – identified by SignalP 3.0 [46] – and their relative abundance, we suggest that these ESTs may encode secreted venom components. In fact, 3 of these clusters encode peptides already found in the venom of *H. gertschi*, which are currently being studied by our group [24 and unpublished].

#### α-KTxs

**Figure 4:**
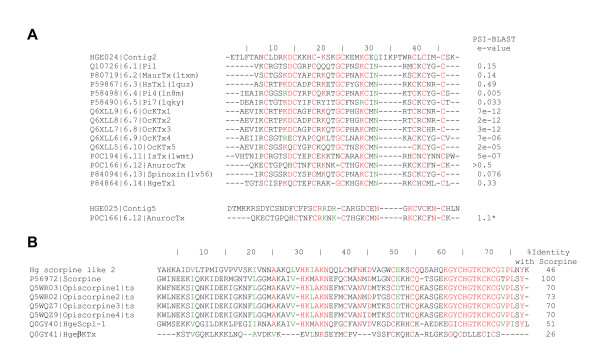
**Scorpion toxin-like precursors in *H. gertschi *venom gland library**. A) Predicted amino acid sequences of the potential α-KTx. HGE024|Contig2 predicted sequence is aligned with all members of the α-KTx 6 subfamily. HGE025|Contig5 is aligned with anuroctoxin (α-KTx 6.12). PSI-BLAST e-values for the third iteration are shown. B) Predicted amino acid sequence of Hg scorpine like 2 and its alignment with others members of the scorpine-like group. The percentage of identity with scorpine is shown. See Supplementary Figure 1 for the complete nucleotide sequences of HGE024|Contig2, HGE025|Contig5 and Hg scorpine like 2. Each sequence starts with its SwissProt accession number followed by common names and Protein Data Bank codes between parentheses (where available). Systematic numbering (sensu [47,49]) for α-KTx is included between accession numbers and common names. Identical amino acids are in red colour and conserved ones in green.

Two clusters encoding potential α-KTx peptides [47-49] were found and their translated sequences are shown in Figure 4 (and Supplementary Figure 1 in Additional file 1). One of those (HGE024|Contig2) is composed by 2 reads, and the other one (HGE025|Contig5) by 17 reads. Although the blastx search against public databases showed that HGE024|Contig2 sequence presents poor e-values (> 10^-5^) with haemocyte defensins of insects and with some OcKTx's – predicted K^+ ^channel toxins from the scorpion *Opistophthalmus carinatus *[50], PSI-BLAST [51] with the translated sequence retrieved most of the members of the α-KTx 6 subfamily (see [1,47-49]) within the first three iterations with good expectance values. Therefore, we propose that HGE024|Contig2 constitutes the precursor of a novel member of this subfamily of K^+ ^channel blockers. Similarly, the search of HGE025|Contig5 sequence against database revealed low similarity with anuroctoxin (α-KTx 6.12) from the scorpion *Anuroctonus phaiodactylus*, a high-affinity blocker of Kv1.3 channels of human T lymphocytes [52]. Again, the expectancy values were rather poor, but in this case, we were already able to purify the mature peptide from the venom of *H. gertschi*, and it is now under study. The prediction of its signal peptide and the N-terminal amino acid sequence determined by automatic Edman degradation (data not shown) reveal that this toxin is in fact produced as a propeptide. This encode for a 67 amino acid-long peptide, containing three segments: an N-terminal signal peptide of 25 amino acid residues, a putative propeptide of 6-amino acids and a mature peptide containing 36 residues (Supplementary Figure 1B in Additional file 1). The mature peptide encoded by HGE025|Contig5 shows several conserved features of α-KTx peptides, nonetheless its sequence is quite unique. We suspect that it might be the first member of a new αKTx subfamily, but this remains to be clarified by ongoing analyses.

#### β-KTx and scorpine-like peptides

One cluster (2 reads) coding the HgeβKTx and another (4 reads) coding the Hge scorpine like were identified in the transcriptome of *H. gertschi *and their sequence have already been reported [24]. Here we present a distinct EST, homologous to the scorpine-like group of long-chain three disulphide-bridged scorpion venom peptides, named Hge scorpine like 2 (Figure 4b and Supplementary Figure 1C in Additional file 1).

#### Cytolytic peptides

**Figure 5:**
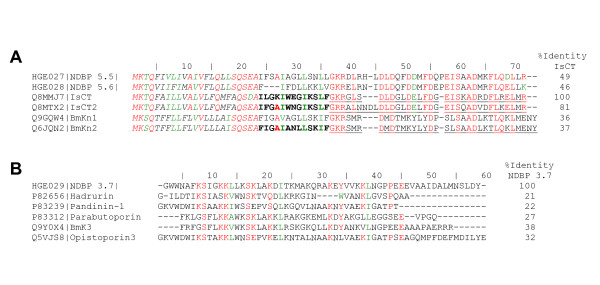
**Predicted amino acid sequences of the novel non-disulfide-bridged peptides (NDBP)**. A) NDBP-5.5 and NDBP-5.6 are aligned with others scorpion cytolytic peptides; the percentage of identity with IsCT is shown. Putative signal peptides are in italics, whereas identified C-terminal prosequences and mature forms are underlined or in bold characters, respectively. B) Alignment of NDBP-3.7 with members of the NDBP 3 subfamily. See Supplementary Figure 2 for the complete nucleotide sequences encoding for NDBP-5.5, NDBP-5.6 and NDBP-3.7. Each sequence starts with its SwissProt accession number followed by common names. Identical amino acids are in red colour and conserved ones in green.

Two clusters encoding IsCT-like precursors were found in *H. gertschi *transcriptome. IsCT and IsCT2 are antimicrobial linear peptides isolated from the scorpion *Opisthacanthus madagascariensis *[53]. They possess broad activity spectra against Gram positive and negative bacteria as well as fungi and relatively weak haemolytic activity against sheep red blood cells. Additionally to the signal peptide, their precursors contain an uncommon acidic propeptide at the C-terminal (Supplementary Figure 2A in Additional file 1). Figure 5a shows both IsCT-like translated sequences, the more abundant comprising 7 reads and the other one represented by 2 reads, classified as non-disulfide-bridged peptides (NDBP) NDBP-5.5 and NDBP-5.6, respectively (following the nomenclature rules of [7]).

#### Bradykinin-potentiating peptide like

One cluster (HGE029|NDBP_3.7, 2 reads; see Supplementary Figure 2B in Additional file 1) encodes a homologue of the bradykinin-potentiating peptide precursor (BmK3 or BmKpbb) from the scorpion *Mesobuthus martensii*, classified as NDBP-3.3 [7]. The angiotensin-bradykinin system is a central hormonal system for the regulation of blood pressure. The angiotensin-converting enzyme (ACE) converts angiotensin I to angiotensin II and degrades bradykinin. Bradykinin potentiating peptides have been isolated from *Tityus serrulatus *(peptide T [54]) and *Buthus occitanus *(K12 [55]). Peptide T, a 13-amino acid linear peptide, potentiates the contractile activity of bradykinin on isolated smooth muscle, inhibits the hydrolysis of bradykinin by ACE, and enhances the depressor effect of bradykinin on arterial blood pressure in the anesthetized rats [54]. Peptide K12 displays similar bradykinin potentiating activities [55]. BmKbpp was identified from *B. martensii *Karsch by cDNA cloning based on the peptide K12 amino acid sequence [56]. The last 21 residues of C-terminal region of BmKbpp showed 57% similarity with peptide K12. Based on the fact that BmKbpp also exhibits high similarity with parabutoporin and others antimicrobial peptides from scorpions, it was suggested that BmKbpp may be a molecule with a dual-function, and that the BmKbpp precursor may be processed in two alternative ways to produces two different mature molecules: BmKbpp and a peptide with only the C-terminal 21 residues of BmKbpp [7]. Figure 5b shows the bradykinin-potentiating peptide like from *H. gertschi*, here named NDBP-3.7.

#### Phospholipases

**Figure 6:**
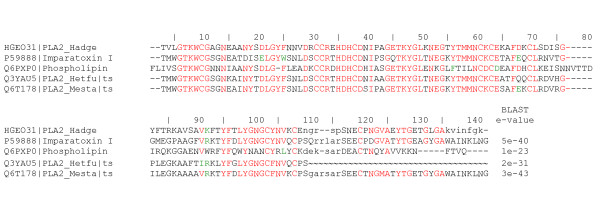
**Putative mature sequence of phospholipase A2 precursor**. Predicted amino acid sequence of *H. gertschi *PLA2 (HGE031|PLA2) aligned with other scorpion venom PLA2. BLAST e-values are shown. See Supplementary Figure 3 for the nucleotide sequence of HGE031|PLA2. Each sequence starts with its SwissProt accession number followed by common names. Identical amino acids are in red colour and conserved ones in green.

A cluster (8 reads) of a new homologue of scorpion venom phospholipases A2 (ScpPLA2) was identified in the *H. gertschi *library (see Supplementary Figure 3 in Additional file 1). The mature form of ScpPLA2 are composed by two subunits, the large ones consisting of approximately 105 amino acid residues, whereas the small subunits have between 18 and 27 residues; their heterodimeric form is stabilized by one interchain disulphide bridge [5,6]. The ScpPLA2 are expressed from a single message, from which the N-terminal propeptide, a penta or hexapeptide internal segment and a short C-terminal region are excised to give the heterodimeric mature form of the enzyme. In Figure 6, these regions are identified on the sequence of the predicted *H. gertschi *PLA2; the assignment was based on multiple sequence alignment of known ScpPLA2. PLA2s are enzymes that catalyze the hydrolysis of the *sn*-2 acyl bonds of *sn*-3 phospholipids, and are normal cellular mediators involved in different responses, such as inflammation, blood hemostasis and others. Many animal venoms posses PLA2s that mediate several toxic responses, like cytotoxicity, neurotoxicity, myotoxicity, edema and blood coagulation disturbs. Based on their primary structure, these toxins can be classified in tree distinct classes: class I is found in Elapidae snakes venom; class II is found in the Viperidae family of snakes; and class III that was identified for the first time in the bee venom. Latter they were found in other invertebrates such as jellyfish, marine snails, and scorpion venoms, but they are also present in vertebrates, like the venomous lizard *Heloderma *[57].

#### Kunitz-type carboxypeptidase inhibitor

**Figure 7:**
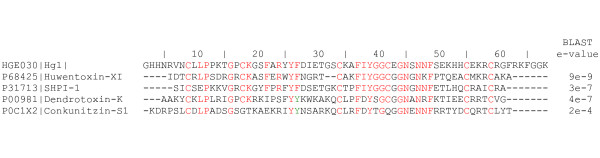
**Multiple sequence alignment of the KU-type proteins of venomous organisms**. Predicted amino acid sequence of HGE030|Hg1 aligned with other venom-derived members of the Kunitz-type serine proteinase inhibitors. BLAST e-values with P68425 (spider *Ornithoctonus huwena*), P31713 (sea anemone *Stichodactyla helianthus*), P00981 (snake *Dendroaspis polylepis polylepis*) and P0C1X2 (cone snail *Conus striatus*) are shown. See Supplementary Figure 4 for the complete nucleotide sequence of HGE030|Hg1. Each sequence starts with its SwissProt accession number followed by common names. Identical amino acids are in red colour and conserved ones in green.

One EST (HGE030|Hg1 (Supplementary Figure 4 in Additional file 1), is homologous to KOG4295, which contains serine proteinase inhibitors of the Kunitz type (KU family). Proteins with KU have been identified in several venomous organisms, like snakes [58], sea anemones [59], cone snails [60] and spiders [61]. However, this is the first report of a KU-type precursor in scorpions. Figure 7 shows the multiple sequence alignment of HGE030|Hg1 with other KU-type venom components. Although the precise role of HGE030|Hg1 in the context of scorpion venom remains to be determined – whether it display neurotoxic or proteinase inhibitor activity –, the ubiquitous presence of proteinase inhibitors suggest a common trend in venomous organisms, deserving further studies.

## Conclusion

Gene cloning of animal toxins has been extensively performed by PCR method, using primers deduced from direct protein sequencing, usually by Edman degradation or mass spectrometry analysis. These studies are aimed at the isolation of specific active components. However, this approach is not entirely suitable for search of unforeseen components that could be present in the venomous gland under study. The strategy is biased by the fact that only those genes that are sharing sequence similarities are usually discovered by this technique. For this reason, we adopted the molecular approach of generating and analyzing ESTs from the *H. gertschi *venom gland as the strategy to produce a general overview of the venom gland transcriptome. This strategy confirms the highly specialized nature of scorpion venom glands as toxin-producer, allowing the description, for the first time, of putative proteins that certainly are involved in cellular processes relevant for the venom glands' function. Additionally, the unguided mining also reveals novel predicted venom components, highlighting the usefulness of the transcriptome approach to improve venom profiling.

## Methods

### cDNA library construction

A cDNA library was constructed from total RNA extracted from a single telson of a *H. gertschi *scorpion. The scorpion was milked 5 days before RNA extraction. For RNA isolation the 'Total RNA Isolation System' of Promega (Madison, WI) was used. With this material a full-length cDNA phagemid library was prepared using the SMART cDNA Library Construction Kit (CLONTECH Lab., Palo Alto, CA). The titre of the non-amplified cDNA library obtained was 2.8 × 10^5 ^cfu/mL with 99% recombinant clones. For the PCR the oligonucleotides TriplEx2-5' (from CLONTECH Lab, Palo Alto, CA) and CD3/3' (5'-AAT CTA GAG GCC GAG GCG GCC GAC ATG-3') [24], designed on the basis of the CDSIII/3' sequence tag used for library construction, were used as primers.

### DNA sequencing and bioinformatic analyses

Selected plasmids were isolated according to a standard alkaline lysis protocol, and single-pass sequencing of the 5'-termini was conducted with the primer TriplEx2-5' (CLONTECH Lab, Palo Alto, CA) using an automatic machine (Model 3100, Applied Biosystems, Foster city, CA) according to the manufacturer's instructions. The nucleotide sequences obtained in this work are deposited in dbEST (GenBank: EL698878–EL698945). To extract the high quality sequence region, the ESTs were subjected to the Phred program [62] with the window length set to 100 and the standard quality to 20. The CrossMatch program was used to remove vector and *E. coli *DNA sequences. ESTs that shared an identity of > 95 out of 100 nucleotides were assembled in contiguous sequences with the CAP3 program [63]. All these bioinformatics analysis were simultaneously run at [64] using default setup. *H. gertschi *venom gland ESTs (clusters and singlets) were searched against nr database [65] using blastx and blastn algorithms [66] with an e-value cutoff set to < 10^-5 ^to identify putative functions of the new ESTs. The signal peptide was predicted with the SignalP 3.0 program [46]. Multiple sequences alignments were obtained using T-COFFEE [67] or CLUSTAL_X [68]. The pairwise identities were calculated with BioEdit [69]. In order to evaluate the most expressed ESTs, a less stringent alignment using a shared identity of > 65 out of 100 nucleotides was used.

With the aim of providing more useful sequence annotations for comparative studies, we selected to identify the eukaryotic orthologous group [38] to which each sequence would belong, instead of reporting the – most often used – highest scored BLAST hit. The best match for each sequence with KOG database was identified by Kognitor – a BLAST-based web server that search within KOGs only – and validated by independent PSI-BLAST searches [51]. The rationale followed was that PSI-BLAST should return all members of the given KOG within the first two iterations. Furthermore, in order to improve sequence annotation and to gain insights into the cellular processes where each sequence could be involved, the ESTs were submitted to GOblet [40] and AmiGO [39] web servers. The aim of this procedure was to identify relevant Gene Ontology terms. Both programs perform blastx searches against UniProt (GOblet) and all GO-annotated protein databases (AmiGO). Cross-validation with identified KOGs was performed by inspection of the retrieved hits in order to identify homologues of the corresponding KOGs. Subsequently the GenBank entries of the given KOG were used as query for AmiGO. GO terms were assigned only when the novel sequence and at least one sequence of each KOGs retrieved coincident hits (usually five or more out of the ten highest-scoring hits).

## Authors' contributions

EFS and EDG designed and carried out most of the experiments described, with some technical assistance (see acknowledgements). EFS and RCRV analyzed the data and wrote the initial draft of the manuscript. LDP supervised the entire project and revised the final version of the manuscript. All authors read and approved the manuscript.

## Supplementary Material

Additional File 1 Supplementary figures. Four figures in a PDF document, including nucleotide and translated sequences of putative venom components. Click here for file

Additional File 2 dbEST submission. Complete submission to the dbEST database in raw text format. Each entry includes: cluster size and, where applicable, assigned eukaryotic orthologous group and relevant Gene Ontology terms. Click here for file
