# Supplementary material for: Transcriptome analysis of the venom gland of the Mexican scorpion Hadrurus gertschi (Arachnida: Scorpiones)
Source: BMC Genomics. 2007 May 16;8:119. doi: 10.1186/1471-2164-8-119 (PMC1904202; doi:10.1186/1471-2164-8-119)
Supplement: Additional File 1 — Supplementary figures. Four figures in a PDF document, including nucleotide and translated sequences of putative venom components. [file 1471-2164-8-119-S1.pdf]

A

```
1     ATG AAT ACA AAA GTT GTC TTA ATC ATG TTG ATG ATC ACT TCA GTG   45
1      M   N   T   K   V   V   L   I   M   L   M   I   T   S   V    15

46    ATT CTG GTT GTT GAA GCA GAA ACC CTT TTT ACT GCC AAT TGC CTC   90
16     I   L   V   V   E   A   E   T   L   F   T   A   N   C   L    30

91    GAT CGC AAA GAT TGT AAA AAA CAT TGC AAA TCT AAA GGA TGT AAG   135
31     D   R   K   D   C   K   K   H   C   K   S   K   G   C   K    45

136   GAA ATG AAA TGC GAG CAA ATA ATT AAA CCA ACT TGG CGT TGT CTC   180
46     E   M   K   C   E   Q   I   I   K   P   T   W   R   C   L    60

181   TGT ATC ATG TGC AGT AAA TAA GACACCCTAACAACTCCTTGCAATATTAATA   225
61     C   I   M   C   S   K  stop

226   TGTACCGGTGTTAAAAAGAATTGAATAAAGAATATTGTTAGC-polyA 3´
```

B

```
1     ATG AAA AAT ATC GCT ATG AAG ACC ACT GTT GTT CTC ACA ATT TTA   45
1      M   K   N   I   A   M   K   T   T   V   V   L   T   I   L    15

46    CTC CTA AGT GTC TTA ACT GCA ATT AAC GCC GAT ACA ATG AAG AAG   90
16     L   L   S   V   L   T   A   I   N   A   D   T   M   K   K    30

91    CGT TCG GAT TAC TGC TCA AAT GAT TTT TGC TTC TTT AGT TGT CGC   135
31     R   S   D   Y   C   S   N   D   F   C   F   F   S   C   R    45

136   CGT GAC CGT TGC GCT AGA GGA GAT TGT GAG AAT GGC AAA TGC GTA   180
46     R   D   R   C   A   R   G   D   C   E   N   G   K   C   V    60

181   TGC AAA AAT TGT CAT TTG AAT TAA AGCCATCCGTTTGGGACGACTCGTCCGT  225
61     C   K   N   C   H   L   N  stop

226   CTCTGGATATTGAAATAAAGTATAGCTTACGT-polyA 3´
```

C

```
1     ATG AAG CTT ACA ATT TTA ATT TTA CTA GTG ATT ACA TCG TTT TGC   45
1      M   K   L   T   I   L   I   L   L   V   I   T   S   F   C    15

46    TCT TGT GGT ATT CTT CGT GAA AAA TAT GCT CAC AAA GCA ATT GAT   90
16     S   C   G   I   L   R   E   K   Y   A   H   K   A   I   D    30

91    GTT TTA ACC CCA ATG ATA GGA GTA CCG GTA GTA TCA AAA ATT GTG   135
31     V   L   T   P   M   I   G   V   P   V   V   S   K   I   V    45

136   AAC AAT GCA GCA AAA CAA TTG GTG CAC AAG ATC GCT AAA AAT CAA   180
46     N   N   A   A   K   Q   L   V   H   K   I   A   K   N   Q    60

181   CAA CTA TGC ATG TTT AAT AAA GAT GTT GCA GGA TGG TGC GAA AAG   225
61     Q   L   C   M   F   N   K   D   V   A   G   W   C   E   K    75

226   AGT TGC CAG CAA TCT GCT CAC CAG AAG GGA TAT TGC CAT GGA ACT   270
76     S   C   Q   Q   S   A   H   Q   K   G   Y   C   H   G   T    90

271   AAA TGC AAA TGT GGC ATT CCA TTG AAC TAC AAA TAA AAACCGAATCA   315
91     K   C   K   C   G   I   P   L   N   Y   K  stop

316   CCATTAAATTCCTCATTTGCTTCAACTTGTTGTTACGTACTGCATGGAAATAATATGAT   360
361   ATCATTAGTAAATAAAACTTTCTCTGTTC
```

Supplementary Figure 1 - Nucleotide and predicted amino acid sequences of novel *H. gertschi* KTx-like precursors.

Nucleotide and predicted amino acid sequences of novel *H. gertschi* KTx-like precursors. Full ESTs and predicted amino acid sequences of putative α-KTxs (A, HGE024|Contig2; B, HGE025|Contig5) and β-KTx (C, HGE026|Hgscplike2). The predicted signal peptides are in italics and the putative poly(A) signals are double-underlined. The putative pro region of HGE025|Contig5 is underlined, whereas its mature sequence is in bold characters.

A

```
NDPB-5.5 1     ATG AAG ACT CAA TTT ATC GTC TTG ATC GTA GCT ATT GTA TTC CTG   45
NDPB-5.6 1     ATG AAA ACT CAA GTC ATC ATC TTT ATC ATG GCA GTT GTA TTC TTG   45

NDPB-5.5 1      M   K   T   Q   F   I   V   L   I   V   A   I   V   F   L    15
NDPB-5.6 1      M   K   T   Q   V   I   I   F   I   M   A   V   V   F   L    15

NDPB-5.5 46    CAA CTT CTT TCC CAA TCA GAA GCC ATC TTT AGT GCA ATT GCT GGT   90
NDPB-5.6 46    CAA TTG CTT TCC CAG TCA GAA GCC TTT --- --- --- ATT TTT GAC   81

NDPB-5.5 16     Q   L   L   S   Q   S   E   A   I   F   S   A   I   A   G    30
NDPB-5.6 16     Q   L   L   S   Q   S   E   A   F   -   -   -   I   F   D    27

NDPB-5.5 91    CTT TTG TCT AAT TTG TTG GGG AAA AGA GAT CTC AGA CAT TTA GAT   135
NDPB-5.6 82    CTT TTG AAG AAA CTC GTT GGG AAA AGA GAA CTC AGA AAT ATA GAT   126

NDPB-5.5 31     L   L   S   N   L   L   G   K   R   D   L   R   H   L   D    45
NDPB-5.6 28     L   L   K   K   L   V   G   K   R   E   L   R   N   I   D    42

NDPB-5.5 136   TTG GAT CAG TTT GAC GAC ATG TTT GAT CAA CCC GAA ATA TCA GCT   180
NDPB-5.6 127   TTA GAT CAG TTT GAC GAC ATG TTT GAT GAA CCC GAA ATA TCA GCT   171

NDPB-5.5 46     L   D   Q   F   D   D   M   F   D   Q   P   E   I   S   A    60
NDPB-5.6 43     L   D   Q   F   D   D   M   F   D   E   P   E   I   S   A    57

NDPB-5.5 181   GCT GAT ATG AAG TTC CTG CAA GAT CTA CTG AGA TAG AAAAAGCATAC   228
NDPB-5.6 172   GCT GAC ATG AGA TTC CTG CAA GAA CTG CTG AAG TAG AACGTGCATAC   219

NDPB-5.5 61     A   D   M   K   F   L   Q   D   L   L   R  stop
NDPB-5.6 58     A   D   M   R   F   L   Q   E   L   L   K  stop

NDPB-5.5       AATCTGTAACACGAGAATTTATTTTGAATCCTAACAATGTAATGGTTGTCCAGTTTTGT
NDPB-5.6       ATACAATATGTGATATGAGAATTTATTTTCCATCATAAGAATTTAATGGTTGTGCAGTT

NDPB-5.5       CAGACATTTCTCGAGTTCTGTGGGATTGGAATAAAATTCAAAATATTAATT-polyA 3´
NDPB-5.6       TAATGGATATTTGTCATCTTCTATTTGATGTCTTAATAAAATCTGAAATCCC-polyA 3´
```

B

```
1      ATG AAT GCC AAA GCC TTT CTA GCT ATT TTC ATG ATT GCA CTA TTA   45
1       M   N   A   K   A   F   L   A   I   F   M   I   A   L   L    15

46     ATT ACA GAT CGA GCT GAA GCA GGC TGG TGG AAT GCG TTT AAA TCC   90
16      I   T   D   R   A   E   A   G   W   W   N   A   F   K   S    30

91     ATA GGA AAG AAA TTA CTG AAA TCA AAA CTT GCC AAG GAT ATT ACA   135
31      I   G   K   K   L   L   K   S   K   L   A   K   D   I   T    45

136    AAA ATG GCA AAA CAA AGA GCA AAA GAG TAT GTT GTA AAA AAG TTG   180
46      K   M   A   K   Q   R   A   K   E   Y   V   V   K   K   L    60

181    AAT GGT CCT CCT GAA GAA GAA GTA GCA GCA ATT GAT GCT CTC ATG   225
61      N   G   P   P   E   E   E   V   A   A   I   D   A   L   M    75

226    AAT TCT TTG GAT TAC TAA GAAAACAAAATATCATCTTATTATTATCACGTTTT
76      N   S   L   D   Y  stop

       CCTTTCACCATATTATAAAATAAATTATTTTGCAACAACAAAC-polyA 3´
```

Supplementary Figure 2 - Nucleotide and predicted amino acid sequences of precursors of *H. gertschi* non-disulphide-bridged peptides.

Complete ESTs and predicted amino acid sequences of HGE027|NDBP_5.5 and HGE028|NDBP_5.6 (panel A), as well of HGE029|NDBP_3.9 (panel B). The predicted signal peptides are in italics and the putative poly(A) signals are double-underlined.

```
1     ATG AGT TTG ATA ATC GTA CTC GTT ATC AGC GTA TTA TCT GCG GAT   45
1      M   S   L   I   I   V   L   V   I   S   V   L   S   A   D    15

46    GCT GTT CTG AGT ATG GAC AAC GAA CTT TAT CTA AAT CTT GAG CCA   90
16     A   V   L   S   M   D   N   E   L   Y   L   N   L   E   P    30

91    AGT CAA CGA AGT AGT TGG CCC GTG GCA AGA GCG GTC CGG ATG CAG   135
31     S   Q   R   S   S   W   P   V   A   R   A   V   R   M   Q    45

136   TTC TCC AAG AGA TCT GAA GGG GGA AGA GAA TCT AGA AAA ATG CAA   180
46     F   S   K   R   S   E   G   G   R   E   S   R   K   M   Q    60

181   GGA TGT CAA ATT CTG GAG TCA CTA AAT GAT ATT GCG AGA GAA GCA   225
61     G   C   Q   I   L   E   S   L   N   D   I   A   R   E   A    75

226   CTT CGC ACT CCA AGA CAC ACC ACG AAG AGA ATA TCT AAA GAC GAA   270
76     L   R   T   P   R   H   T   T   K   R   I   S   K   D   E    90

271   ATG GAA TTC TTT GAA GGA AGA TGC CTC AGC GTA GGA GAA TCA GAA   315
91     M   E   F   F   E   G   R   C   L   S   V   G   E   S   E    105

316   CGT ACA GTT TTG GGG ACA AAG TGG TGT GGC GCT GGA AAC GAA GCA   360
106    R   T   V   L   G   T   K   W   C   G   A   G   N   E   A    120

361   GCA AAT TAC TCG GAT CTT GGC TAT TTT AAC AAT GTT GAT CGC TGC   405
121    A   N   Y   S   D   L   G   Y   F   N   N   V   D   R   C    135

406   TGC CGT GAA CAC GAT CAC TGC GAT AAT ATT CCA GCG GGA GAA ACT   450
136    C   R   E   H   D   H   C   D   N   I   P   A   G   E   T    150

451   AAA TAT GGC CTG AAG AAC GAA GGA ACA TAT ACC ATG ATG AAT TGC   495
151    K   Y   G   L   K   N   E   G   T   Y   T   M   M   N   C    165

496   AAA TGT GAG AAG GCG TTT GAT AAA TGT TTG AGC GAC ATT TCT GGA   540
166    K   C   E   K   A   F   D   K   C   L   S   D   I   S   G    180

541   TAC TTT ACA AGA AAA GCT GTT TCT GCT GTC AAA TTT ACA TAT TTT   585
181    Y   F   T   R   K   A   V   S   A   V   K   F   T   Y   F    195

586   ACA CTT TAT GGA AAT GGC TGC TAC AAC GTC AAA TGC GAG AAT GGA   630
196    T   L   Y   G   N   G   C   Y   N   V   K   C   E   N   G    210

631   AGA TCC CCA AGC AAT GAA TGT CCC AAC GGT GTG GCG GAA TAT ACG   675
211    R   S   P   S   N   E   C   P   N   G   V   A   E   Y   T    225

676   GGT GAA ACG GGA CTT GGT GCT AAG GTG ATA AAT TTC GGA AAG TGA   720
226    G   E   T   G   L   G   A   K   V   I   N   F   G   K  stop  240

      AGTATTTGCCAGTCGAAAAACAGACAGTATTTTATAAATACGTCGATTTCAAAATATGT
      GCTATTAATTCTTGTACTTGAAATAAGAATGAAATAAAGGAACAAAATAAATT-polyA 3´
```

Supplementary Figure 3 - Nucleotide and predicted amino acid sequences of the *H. gertschi* heterodimeric PLA2 (HGE031|PLA2).

The predicted signal peptide is in italic and the putative poly(A) signal is double-underlined. The assignment of putative pro regions (underlined) and mature sequence (in bold) was done based on multiple sequence alignment of known ScpPLA2.

1	ATG	ATT	ATC	TTT	TAC	GGT	CTT	TTC	TCT	ATT	TTA	GTT	CTC	ACT	TCC	45
1	*M*	*I*	*I*	*F*	*Y*	*G*	*L*	*F*	*S*	*I*	*L*	*V*	*L*	*T*	*S*	15
46	ATC	AAC	ATT	GCG	GAA	GCA	GGC	CAT	CAT	AAC	AGA	GTT	AAC	TGC	CTG	90
16	*I*	*N*	*I*	*A*	*E*	*A*	G	H	H	N	R	V	N	C	L	30
91	CTT	CCG	CCA	AAA	ACA	GGC	CCT	TGT	AAA	GGT	TCC	TTT	GCA	CGA	TAC	135
31	L	P	P	K	T	G	P	C	K	G	S	F	A	R	Y	45
136	TAT	TTT	GAT	ATC	GAA	ACG	GGA	AGT	TGC	AAA	GCA	TTC	ATA	TAT	GGA	180
46	Y	F	D	I	E	T	G	S	C	K	A	F	I	Y	G	60
181	GGA	TGT	GAA	GGG	AAT	TCC	AAT	AAT	TTC	TCA	GAA	AAG	CAT	CAT	TGT	225
61	G	C	E	G	N	S	N	N	F	S	E	K	H	H	C	75
226	GAA	AAG	AGA	TGC	CGA	GGT	TTT	CGA	AAG	TTT	GGC	GGC	AAA	TAA	ATT	270
76	E	K	R	C	R	G	F	R	K	F	G	G	K	stop		

271 CTATGGTATAAAGACAAAGCATCAACAATTTCCATTAAATAAATTTTTTACTTACTCCT-polyA 3´

Supplementary Figure 4 - Nucleotide and predicted amino acid sequences of *H. gertschi* KU-type precursor HGE030|Hg1.

The predicted signal peptide is in italics and the putative poly(A) signal is double-underlined.
